# Two distinct penicillin binding proteins promote cell division in different *Salmonella* lifestyles

**DOI:** 10.15698/mic2018.03.622

**Published:** 2018-02-17

**Authors:** Sónia Castanheira, Juan J. Cestero, Francisco García-del Portillo, M. G. Pucciarelli

**Affiliations:** 1 Laboratorio de Patógenos Bacterianos Intracelulares. Centro Nacional de Biotecnología-Consejo Superior de Investigaciones Científicas (CNB-CSIC), Madrid, Spain.; 2 Centro de Biología Molecular Severo Ochoa-Consejo Superior de Investigaciones Científicas (CBMSO-CSIC), Madrid, Spain.; 3 Departamento de Biología Molecular, Universidad Autónoma de Madrid, Madrid, Spain.

**Keywords:** Salmonella, division, PBP3, PBP3SAL, acidic pH, intracellular, phagosome

## Abstract

The bacterial cell wall preserves cell integrity in response to external insults and the internal turgor pressure. The major component of the cell wall is the peptidoglycan (PG); a giant macromolecule formed by glycan chains cross-linked by short peptides. The PG is synthesized by a stepwise process that includes cytosolic and periplasmic reactions. The building subunits -muropeptides- are incorporated into the growing macromolecule by transglycolyslation (TG) and transpeptidation (TP) reactions, which constitute the last biosynthetic steps. TP reactions, involving cleavage of the terminal D Ala-D-Ala bond in the stem peptide, are carried out by enzymes known generically as penicillin-binding proteins (PBPs) due to their capacity to bind β lactam antibiotics, which are D Ala-D-Ala structural analogues. On an average, bacterial genomes harbour a minimum of 10 PBP-encoding genes, most of them non-essential. This dispensability has led to the widely accepted concept of functional redundancy for many PBPs. An exemption is the PBP dedicated to build the septal PG required to separate daughter cells during cell division. To date, this division specific PBP was reported as unique in all known bacteria and, as a consequence, “essential”. Our recent results obtained in the intracellular bacterial pathogen *Salmonella enterica* serovar Typhimurium challenges this view since this bacterium has two PBPs that can independently build the division septum. One of these two division PG enzymes is orthologue of the division-specific PBP3 of *Escherichia coli*. The second enzyme, named PBP3_SAL_, is absent in non-pathogenic bacteria and, at least in *S.* Typhimurium, displays PG biosynthetic activity restricted to acidic conditions. Our work also revealed that it is possible to generate a *S.* Typhimurium mutant defective in PBP3, which cannot divide at neutral pH.

*Salmonella* *enterica* serovar Typhimurium (*S. *Typhimurium) is an intracellular bacteria pathogen that invades and proliferates inside phagocytic and non-phagocytic eukaryotic cells. Although recent studies provided evidence for the capacity of this pathogen gaining access to the cytosol of certain host cell types, in most cases the intracellular bacteria reside within acidic phagosomes. These acidic compartments are strikingly different compared to the nutrient media in which bacteria are usually grown in the laboratory, which are buffered to neutral pH to favour optimal growth. When adapted to the intra-phagosomal lifestyle, *S.* Typhimurium exhibits lower growth rate than in the cytosol or the extracellular milieu. Whether *S.* Typhimurium utilizes distinct machinery for synthesis and remodelling of the cell envelope in these two different lifestyles (extra- versus intracellular) is mostly unknown, especially for its main component, the peptidoglycan (PG). Altered PG structure linked to the colonization of the intracellular niche has important implications for host defences that evolved to recognize PG fragments in an environment, normally not colonized by bacteria, e.g. the eukaryotic cytosol. Members of this defence mechanism include the PG-sensing receptors NOD1 and NOD2. We reported recently the induction in intracellular *S.* Typhimurium of an PG enzyme with D-L-endopeptidase activity that is absent in non-pathogenic bacteria and cleaves the D-glutamic acid(D-Glu)-*meso*-diamopimelic acid (*m*Dap) bond of the PG stem peptide. This D-Glu-*m*Dap motif is absolutely essential for NOD1 recognition. The activity of this D-L-endopeptidase could be therefore exploited by intracellular *S.* Typhimurium to minimize NOD1 recognition.

Our comparative genome analyses, directed to identify *Salmonella*-specific genes encoding new PG enzymes, revealed that, besides the *ftsI* gene encoding the PBP3 involved in cell division, the *S.* Typhimurium genome has a gene encoding a PBP3 paralogue (63% identity at the amino acid level). This PBP3 paralogue, which we named PBP3_SAL_, is also a class B PBP with a transpeptidase (TP) domain in which the three catalytic motifs, SXXK (containing the active serine site), SXN/D and KTG, are conserved. Binding assays based on fluorescent β lactam antibiotics (Boc-FL) demonstrated that PBP3_SAL _is a genuine PBP. Interestingly, our initial experiments showed that, unlike PBP3, the PBP3_SAL_ was able to bind Boc-FL "exclusively" at acidic pH. This observation agreed with the fact that PBP3_SAL_ restored cell division at 42°C in an *E. coli* strain producing a thermosensitive PBP3 only when bacteria were grown in acidified medium. Altogether, these results indicated that PBP3_SAL _evolved for promoting cell division in acidic environments.

Additional experiments sustained a role of PBP3_SAL_ as an enzyme promoting cell division during the interaction of *S.* Typhimurium with the mammalian host. We were successful in the generation of a *S.* Typhimurium mutant lacking the hitherto considered essential PBP3 and that showed striking virulence phenotypes. This *ftsI *null mutant was capable of growing and dividing inside phagosome of cultured eukaryotic cells, with rates even higher than those of wild type bacteria. In the *in vivo* experiments using the mouse typhoid model, this Δ*ftsI *mutant was however attenuated, which we explained by its requirement for acidic pH to divide. The spread of the pathogen throughout host cells and tissues takes place by alternate episodes in which bacteria locate extra- and intracellularly. Any long permanence of the Δ*ftsI *mutant outside cells would be fatal for its capacity to invade cells. Thus, this mutant in the neutral extracellular space continues elongating but without dividing. This could result in long filamentous bacteria that could be impeded to invade a neighbour cell and, as consequence, to initiate a new intracellular infection cycle.

Two sets of additional data point to the necessity of studying more in detail the function of PBP3_SAL_. On one side, PBP3_SAL_ binds poorly some β-lactam antibiotics that display high affinity for PBP3. An example is cefuroxime, which is of wide clinical use. On the other hand, the mouse typhoid model revealed that PBP3_SAL_ is produced predominantly *in vivo* by *S. *Typhimurium during the colonization of target organs such as spleen. Therefore, PBP3_SAL_ is certainly produced in the host environment and, given its strict dependence of acidic pH for activity, it must necessarily be produced along the intracellular infection. The basis of this different affinity for antibiotic binding is still unknown, although it probably may reflect distinct natural substrates used for their contribution to PG biosynthesis.

The presence of two PBPs capable of promoting "alone" cell division in *S. *Typhimurium is intriguing. There is only partial function redundancy, since our data proved that one of them, PBP3, is more versatile in comparison with the highly specialized PBP3_SAL_. This situation in *S. *Typhimurium differs respect a case recently reported in *Bacillus subtilis*, in which PBP3 -a "dispensable" PBP in this organism- could promote cell division in a mutant expressing a catalytic-death variant of the cell-division specific PBP2B. The authors of this work discussed about a "structural role" of *B. subtilis *PBP2B in the division machinery that could be independent of its biosynthetic role in incorporating muropeptides to the division septum. Unlike the case of *S. *Typhimurium, in which the genes of both PBPs (3 and 3_SAL_) can be genetically inactivated by separate, inactivation of PBP2B in *B. subtilis* is lethal. It is worth to note that *B. subtilis* PBP2B of *B. subtilis* shows sequence relation with PBP2A of *Staphylococcus aureus*, responsible for methicillin resistance. The appearance of "PBP variants" could be therefore positively favoured by a necessity to replace the function of housekeeping PBPs in adverse conditions like the exposure to antibiotics, which inactive those PBPs displaying high affinity for them.

In the case of PBP3_SAL_ and PBP3, the rationale of this duplication, at least in terms of a PBP capable of building the division septum, is however not apparent. The up-regulation of PBP3_SAL _in the phagosomal compartment and in animal organs raises the question of why PBP3 is not favoured when the bacteria are enclosed within acidic phagosomes. Considering that our work demonstrates that PBP3 can also perform cell division in acidified laboratory media, our data point to the possibility that the unique acidic phagosomal environment may impair optimal activity of PBP3 in cell division. Future studies should shed light into this tempting idea.

Other aspects deserve our attention in future studies focused on PBP3_SAL_ (**Figure 1**). An example are the yet unknown regulators that induce the expression of PBP3_SAL _*de novo* when the pathogen invades the phagosomal compartment. Several *S.* Typhimurium regulators required for virulence such as the two-component systems PhoP-PhoQ and OmpR EnvZ respond to the acidic phagosomal pH. Work is in progress to dissect whether the up-regulation of PBP3_SAL_ is connected to that of other virulence factors with activity in the phagosomal compartment such as the type III secretion system encoded in the *Salmonella-*pathogenicity island 2 (SPI-2). In addition to regulation, a major point to examine is whether PBP3 and PBP3_SAL_ "co-exist" in the same bacterial cell or, by contrast, are expressed by different individuals of the population. Single-cell based analyses repeatedly reveal that many genes are expressed by bacteria in a rather stochastic manner, a possibility that could be addressed for PBP3_SAL_. At this respect, it is important to note that *ftsI *maps within a large operon having downstream essential genes. So, it will be of much interest to discern how PBP3 can be down-regulated without disrupting much the function of other division-related proteins. Moreover, PBP3_SAL_ does not function in extracellular spaces having neutral pH, so equally important will be to unravel how the PBP3_SAL_ is turned off when the pathogen egresses from the infected cell. Additional important questions to answer in the future include the PG built by intra-phagosomal and cytosolic bacteria. Since PBP3_SAL _is inactive in the neutral pH of the cytosol and, considering the possibility of differential expression of other PBPs in these two population, we can expect structural differences in the PG with probably different consequences in host cell signalling.

**Figure 1:**
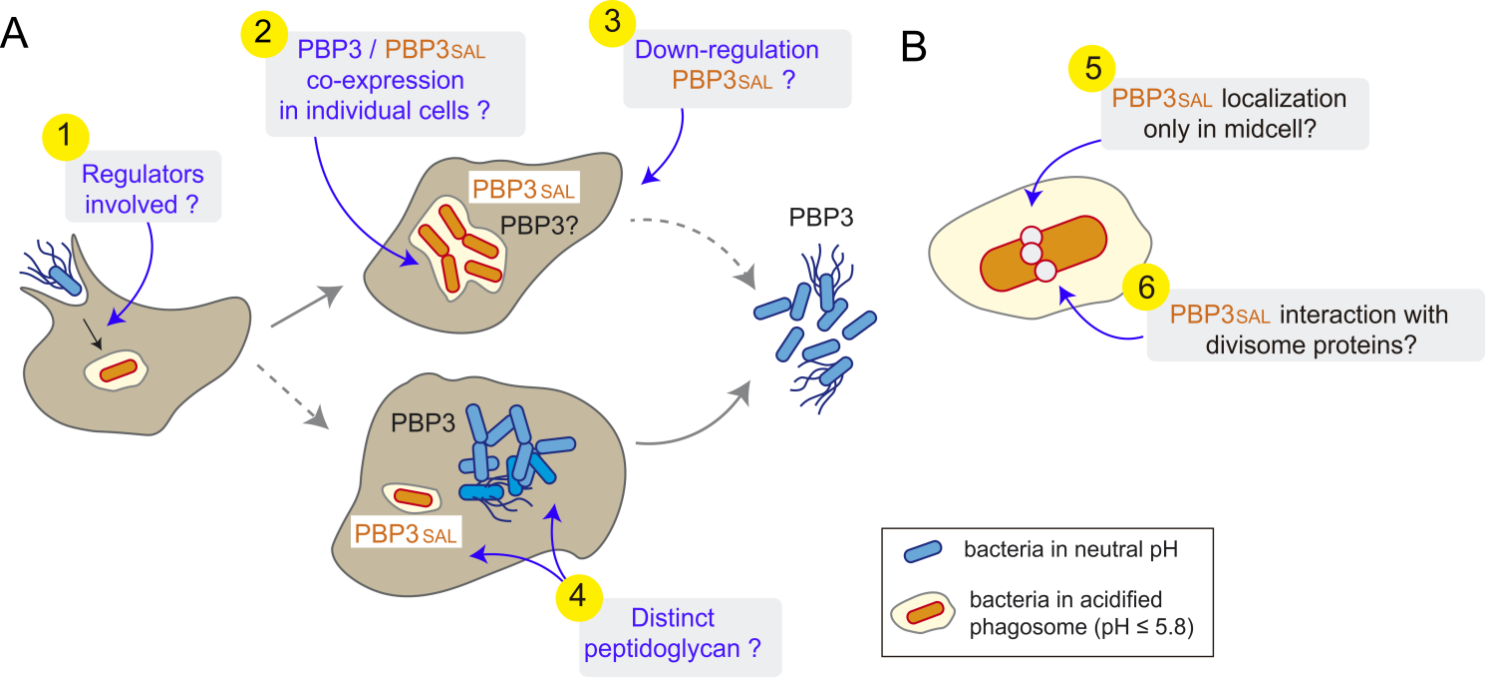
FIGURE 1: Existing gaps in our current knowledge of PBP3_SAL_ function in *S.* Typhimurium. **(A)** Phenomena to be analysed in future studies respect PBP3_SAL_ activity during the interaction of *S. *Typhimurium with the eukaryotic cell. These include: (1) the regulators that upregulate expression of PBP3_SAL_ following entry into the phagosomal compartment; (2) whether intracellular bacteria express PBP3 in the acidic phagosome and, in that case, if it is co-expressed with PBP3_SAL_; (3) how PBP3_SAL_ expression is downregulated when bacteria exit from the infected host cell to the extracellular space; and, (4) given the specialization of PBP3_SAL_ for functioning exclusively in acidic environments, whether there are additional differences in the PG machinery of intra-phagosomal and cytosolic bacterial populations that could result in a PG structural alterations; **(B)** Aspects that at the bacterial cell level that remain unknown for PBP3_SAL_, including: (5) the exact localization of PBP3_SAL _on the bacterial surface; and, (6) the interaction of PBP3_SAL _with divisome proteins required to build a division septum independently of PBP3.

Two features of PBP3_SAL_ at the level of the bacterial cell also need to be commented. On one side, its location on the bacterial surface. Since our work demonstrated that PBP3_SAL_ can promote division in the absence of PBP3, PBP3_SAL_ must be targeted to the mid-cell. However, until this is addressed experimentally using fluorescent reporters with physiological levels of the protein, we cannot rule out other surface locations for PBP3_SAL_. Another intriguing question involves the interaction of PBP3_SAL _with divisome proteins. Does PBP3_SAL_ highjack the divisome machinery that normally assembles to promote PBP3-mediated division? Biochemical approaches involving identification of partners either shared or specific to each of these two PBPs should shed light into these new ideas.

Lastly, it is important to recapitulate on the fact that the *S. *Typhimurium gene encoding PBP3_SAL_ is an islet, flanked by *rlmA* and *cspC*. PBP3_SAL_ orthologue genes displaying a high degree of synteny are also present in genomes of *Citrobacter koseri*,* C. freundii*, *C. rodentium*,* Enterobacter hormaechei*,* E. cloacae*, and *E. cancerogenus*, all pathogenic bacteria. An obvious question is why these pathogens in which the intracellular infection is not prominent, have also a PBP3 paralogue. A similar question arises when considering early work performed in *Pseudomonas aeruginosa*, which identified a non essential PBP3 paralogue for which function was not assigned due to the essential nature of the housekeeping PBP3. It is clear that future work should focus on these probable "redundant" division proteins in otherwise extracellular pathogens. Based on the reference established by our work on PBP3_SAL _of *S. *Typhimurium_, _such analyses could involve characterization of their activity at different pH values, binding assays to distinct β-lactam antibiotics and the usage of genetic tricks to attempt inactivating the endogenous PBP3, hitherto considered essential.

